# Association between gallstones and the risk of biliary tract cancer: a systematic review and meta-analysis

**DOI:** 10.4178/epih.e2021011

**Published:** 2021-02-03

**Authors:** Dan Huang, Hyundeok Joo, Nan Song, Sooyoung Cho, Woosung Kim, Aesun Shin

**Affiliations:** 1 Department of Preventive Medicine, Seoul National University College of Medicine, Seoul, Korea; 2 Division of Cancer Control and Policy, National Cancer Control Institute, National Cancer Center, Goyang, Korea; 3 Seoul National University College of Medicine, Seoul, Korea; 4 Cancer Research Institute, Seoul National University, Seoul, Korea; 5 Department of Epidemiology and Cancer Control, St. Jude Children’s Research Hospital, Memphis, TN, USA

**Keywords:** Gallstones, Biliary tract neoplasms, Gallbladder neoplasms, Bile duct neoplasms, Cholangiocarcinoma, Ampulla of Vater

## Abstract

**OBJECTIVES:**

Biliary tract cancers (BTCs) are rare but highly fatal. Although the etiology of BTC is poorly understood, gallstones are proposed to be a major risk factor. We conducted a systematic review and meta-analysis to examine the associations between gallstone characteristics and BTC risk.

**METHODS:**

We searched the MEDLINE, Embase, and Cochrane Central databases and systematically reviewed cohort and case-control studies published before April 9, 2018. All the included studies reported appropriate risk estimates and confidence intervals (CIs) for associations between the presence, size, number, or duration of gallstones and the risk of BTC, including gallbladder cancer (GBC), extrahepatic bile duct cancer (EBDC), and ampulla of Vater cancer (AOVC). Summary odds ratios (ORs) and their 95% CIs were calculated using a random-effects model in the meta-analysis. Subgroup analyses were conducted to inspect sources of potential heterogeneity, and the Egger test was performed to assess publication bias.

**RESULTS:**

Seven cohort studies and 23 case-control studies in Asian, European, and American populations were included. The presence of gallstones was associated with an increased risk of BTC (OR, 4.38; 95% CI, 3.23 to 5.93; I^2^=91.2%), GBC (OR, 7.26; 95% CI, 4.33 to 12.18), EBDC (OR, 3.17; 95% CI, 2.24 to 4.50), and AOVC (OR, 3.28; 95% CI, 1.33 to 8.11). Gallstone size (>1 vs. <1 cm; OR, 1.88; 95% CI, 1.10 to 3.22) was significantly associated with the risk of GBC.

**CONCLUSIONS:**

Gallstone characteristics, such as presence, size, and number, are associated with an increased risk of BTC. However, significantly high heterogeneity in the meta-analyses is a limitation of this study.

## INTRODUCTION

Biliary tract cancer (BTC) is relatively uncommon in most parts of the world [1], but is classified as a major cancer based on its incidence in certain countries such as the Korea and India [2,3]. An increasing incidence of BTC has been observed at all 3 biliary tract subsites, specifically gallbladder cancer (GBC), extrahepatic bile duct cancer (EBDC), and ampulla of Vater cancer (AOVC), especially in high-risk areas [4,5]. The prognosis of BTC is generally poor, and the estimated 5-year survival rate is only approximately 5% [6]. Although surgery can be curative, only a small percentage of patients are candidates for surgery because a high proportion of patients are diagnosed at a late stage of the disease [7]. To improve the survival rate, early detection of the disease based on the identification of risk factors is important.

Gallstones, concretions formed in the biliary tract, have been suggested as an important risk factor for BTC [5]. The carcinogenic mechanisms of BTC are poorly understood, but they may involve inflammatory changes near stones [8]. BTC could arise as a result of chronic inflammation associated with gallstones continuously irritating the gallbladder and bile duct [9]. While gallstones are a common condition [10], BTC rarely occurs, and most people with gallstones never end up developing cancer [11,12]. However, a significant number of BTC patients have gallstones [13], which leaves room for further investigations into the potential association between gallstones and the risk of BTC. One study attempted a systematic review [14], but it examined the literature on the association between benign gallbladder disease (the broader term used to represent gallstones) and the risk of BTC. There is a scarcity of reviews focusing on the relationship between gallstones and the risk of BTC.

We conducted a systematic review and meta-analysis of published cohort and case-control studies on associations between gallstone characteristics and the risk of BTC. This study aimed to update the latest studies through a systematic review and to provide a better description of the association of gallstones with the risk of BTC, encompassing its known subtypes GBC, EBDC, and AOVC [15], while intentionally excluding intrahepatic bile duct cancer (IBDC) and intrahepatic cholangiocarcinoma. The objective is this study is to synthesize data from the vast populations included in various studies (case-control and cohort studies) throughout the world to determine to what extent patients with gallstones are more likely to develop BTC and each of its subtypes than hospital-based or community-based control groups. In this study, gallstones were characterized in terms of their presence, size, number, and duration, and detailed subgroup analyses were also performed stratified by the study design, sex, geographic area, study period, measurement of exposure, study quality score, and adjustment of confounders.

## MATERIALS AND METHODS

The study protocol followed the PRISMA (Preferred Reporting Items for Systematic Reviews and Meta-Analysis) guidelines.

### Data sources and searches

The first and second reviewers (DH and HJ) searched the PubMed, Embase and Cochrane Library databases for epidemiological studies with the following keywords: (“gallstone” OR “calculi” OR “cholelithiasis” OR “cholecystolithiasis” OR “choledocholithiasis”) AND (“biliary tract cancer” OR “biliary tract neoplasms” OR “gallbladder cancer” OR “gallbladder neoplasms” OR “gallbladder carcinoma” OR “cholangiocarcinoma” OR “extrahepatic bile duct cancer” OR “extrahepatic cholangiocarcinoma” OR “ampulla of vater cancer”). Medical subject headings (MeSH) terms were used for the PubMed search, and Emtree explode terms were used for the Embase search when available. The last search was conducted on Aug 9, 2018. The language was restricted to English in PubMed and Embase, but not in the Cochrane Library database. In terms of publication status, our search was confined only to published human studies. Papers published before April 9, 2018, were reviewed. Duplicates were excluded, and additional papers obtained by manually searching the references of the selected articles were included.

### Study selection

The inclusion criteria for eligible studies were as follows: (1) cohort or case-control studies on the association between gallstones and the risk of BTC (GBC, EBDC, or AOVC); (2) gallstones (presence, size, number, or duration) as the exposure of interest; (3) studies in which the primary outcome was the occurrence of BTC (GBC, EBDC, or AOVC); and (4) studies that reported risk estimates (rate ratio [RR], odds ratio [OR], or hazard ratio [HR]) and their 95% confidence intervals (CIs). Studies were excluded if any of the following criteria were met: (1) non-human studies; (2) non-observational studies or observational studies without an analytical epidemiologic approach; (3) irrelevant exposure or outcome variables (hepatolithiasis or intrahepatic cholangiocarcinoma); (4) duplication or unobtainable abstract/full-text; (5) the absence of a risk estimate that was either reported or could be calculated by the given information.

### Data extraction

The first and third reviewers (DH and WK) (under the supervision of AS) independently screened the titles and abstracts of studies that met the inclusion criteria. The full texts were reviewed by 2 independent reviewers (DH and HJ) and the supervisor (AS).

The 2 independent reviewers (DH and HJ) extracted data using a standardized extraction form. When discrepancies arose, a fourth investigator (NS) made the final decision for study eligibility and data extraction. The relevant data included the last name of the first author, publication year, study country, study design (cohort or case-control study), study period, sex, sample size (number of cohorts and incident cases for cohort studies or number of cases and controls for case-control studies), exposure variables (presence, size, number, and duration of gallstones), measurement of exposure (with or without imaging studies), outcome variables (occurrence of GBC, EBDC, AOVC), duration of follow-up for cohort studies, adjustment variables in the statistical analysis, and risk estimates, such as OR, RR, and HR with corresponding 95% CIs.

### Quality assessment

Quality assessment data were extracted using the Newcastle-Ottawa scale (NOS), which contains 9 items, with 8 items receiving 1 point and 1 item accounting for 2 points, leading to a maximum of 10 points [16]. A quality score equal to or greater than the median value was judged as indicating high quality.

### Statistical analysis

In this study, the summary risk estimates and their corresponding 95% CIs were calculated using a random-effects model [17]. Selected studies reported different types of risk estimates, such as ORs, RRs, and HRs. RRs and HRs were treated as equivalent to ORs. We compared gallstone characteristics as follows: presence (present vs. absent), size (≥ 1 vs. < 1 cm, ≥ 2 vs. < 2 cm), number (> 1 vs. 1), and duration. For studies reporting multiple risk estimates according to the subsites of BTC (GBC, EBDC, and/or AOVC), the pooled risk estimates and their corresponding 95% CIs that were adequate for meta-analysis were taken as representative risk estimates.

Statistical heterogeneity across studies was appraised using the I^2^ statistic and the chi-square-based Q tests. I^2^ values of 25%, 50%, and 75% indicated low, moderate, and high heterogeneity, respectively [18]. For the Q statistic, a p-value < 0.10 was considered to indicate statistically significant heterogeneity. To perform subgroup analyses, we stratified studies by study design, sex, geographic area (Asia and non-Asia), study period (before, around, and after 2000; around 2000 refers to studies where the starting point was before 2000 but the ending point was after 2000), measurement of exposure, study quality according to the NOS, and whether the analysis adjusted for confounders (such as age, sex, comorbidity, lifestyle factors, education, and/or geographic areas). Sensitivity analyses [19] were conducted by sequentially excluding 1 study at a time to evaluate the influence of individual studies on the stability of the pooled results. Forest plots were used to present results graphically. Publication bias was investigated through funnel plots [20] with the Egger test [21], and p-values < 0.01 indicated statistical significance. All statistical analyses were performed using Stata version 15.0 (StataCorp., College Station, TX, USA). A 2-tailed p-value < 0.05 was considered to indicate statistical significance, except as otherwise specifies.

### Ethics statement

Informed consent was waved due to the study design (systematic review and meta-analysis).

## RESULTS

### Study selection and characteristics

**Figure 1.**
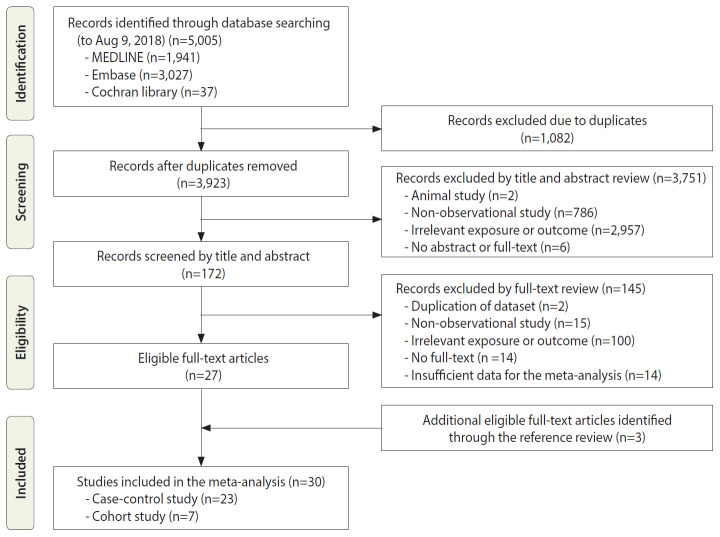
Flow chart of study selection for the meta-analysis.

Figure 1 shows the process of study selection for the meta-analysis. Initially, we retrieved a total of 5,005 articles, including 1,941 from MEDLINE, 3,027 from Embase, and 37 from the Cochrane Library. We excluded 1,082 duplicate studies. Based on reviewing the titles and abstracts, 3,751 other studies were excluded for various reasons: animal studies (n =2); non-observational studies (n=786); irrelevant exposures or outcomes (n=2,957); and no abstract or full text (n=6). We reviewed the full texts of the remaining 172 studies and excluded articles with irrelevant exposures or outcomes, insufficient data for the meta-analysis, or other exclusion criteria, thus resulting in 27 eligible articles. Additionally, 3 relevant studies were included by searching the reference lists of the eligible articles. Thus, a total of 30 epidemiological studies, including 7 cohort studies and 23 case-control studies, were included in this meta-analysis.

The characteristics of the 7 cohort studies [12,22-27] and 23 case-control studies [13,28-49] are shown in Table 1. Of these studies, 16 studies were conducted in Asia, 8 studies were conducted in America, and 6 studies were conducted in Europe. Associations between gallstones and the risk of GBC were investigated in 18 studies, where gallstones were characterized in terms of their presence in 14 studies, their size in 4 studies, and their number in 2 studies. In terms of the risk of EBDC, with the concept of EBDC embracing extrahepatic cholangiocarcinoma (EHC), bile duct cancer (BDC), and cholangiocarcinoma (CCA), there were 17 studies that examined the association between gallstone presence with the risk of BTC. All 5 studies on AOVC reported an association between gallstone presence and the risk of cancer.

### Gallstones and the risk of biliary tract cancer

**Figure 2.**
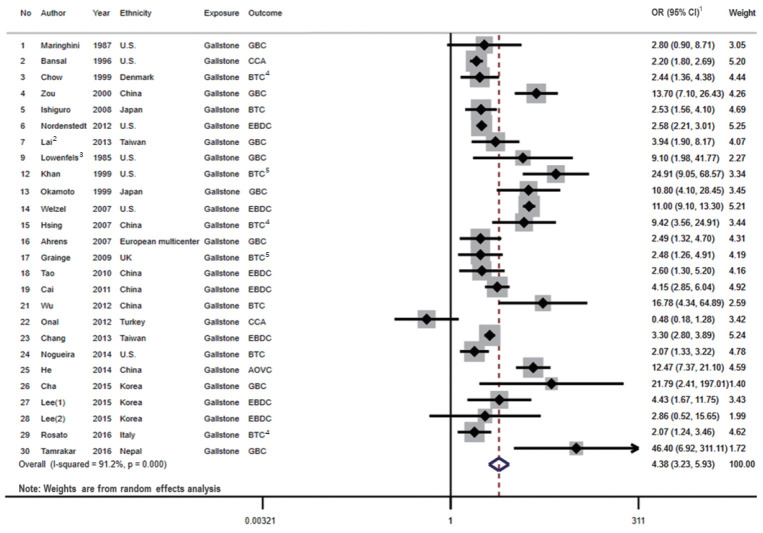
Forest plot showing the relationship between presence of gallstone and the risk of BTC. ^1^OR (95% CI) refers to the estimate of effects included in a random-effects model. ^2^Effect size of Lai et al. [27] was calculated by pooling the results of DM group and non-DM group. ^3^Effect size of Lowenfels et al. [29] was calculated by pooling the results of Indian and non-Indian. ^4^Pooled the results of three types BTC subsites. ^5^Pooled the results of two types BTC subsites. The numbers are arranged in the order of Table 1. OR, odds ratio; CI, confidence interval; GBC, gallbladder cancer; CCA, cholangiocarcinoma; BTC, biliary tract cancer; EBDC, extrahepatic bile duct cancer; AOVC, ampulla of Vater cancer; DM, diabetes mellitus.

A total of 26 studies presented associations between the presence of gallstones and the risk of BTC (Figure 2). Among these studies, only 2 studies referred to BTC specifically, and the remaining 24 studies described the risk estimates according to the subsites of BTC (GBC, EBDC, and/or AOVC).

We identified 7 cohort studies and 19 case-control studies that presented associations between the presence of gallstones and the risk of BTC. When we examined the results stratified by the study design, a statistically significant positive association was shown in both case-control studies (OR, 5.04; 95% CI, 3.36 to 7.56; I^2^=90.5%; p<0.001) and cohort studies (OR, 3.17; 95% CI, 2.28 to 4.39; I^2^=79.0%; p<0.001). The pooled risk estimate was also statistically significant (OR, 4.38; 95% CI, 3.23 to 5.93), with high heterogeneity across the studies (I^2^=91.2%; p<0.001).

In subgroup meta-analyses, all results showed statistical significance, regardless of sex, geographic area, study period, measurement of exposure, study quality, and adjustment for confounders, as shown in Table 2. The magnitudes of the associations were larger in females (OR, 4.26; 95% CI, 2.75 to 6.59; I^2^=84.5%; p<0.001) than in males, larger in Asia (OR, 5.25; 95% CI, 3.50 to 7.86; I^2^=82.4%; p<0.001) than outside of Asia, larger in studies conducted before 2000 (OR, 5.39, CI, 2.57 to 11.34; I^2^=95.5%; p<0.001) than in studies conducted around and after 2000, larger in studies with imaging studies (OR, 7.09; 95% CI, 3.87 to 12.98; I^2^=64.5%; p=0.004) than in studies without imaging studies, and larger in low-quality studies (OR, 4.81; 95% CI, 2.87 to 8.05; I^2^=94.9%; p<0.001) than in high-quality studies. The meta-analysis indicated that the association became weaker after adjusting for age, sex, and comorbidities. A stronger association than the original result was observed after adjusting for geographic areas, lifestyle factors, and education. However, there were no significant differences between the magnitudes of association under any stratifications.

The heterogeneity varied substantially as the stratification method changed, and subgroup analysis with the studies that reported the outcomes of only the male patients exhibited the lowest level of heterogeneity (I^2^=35.8%; p=0.132) among the subgroups including more than 2 studies.

### Gallstones and the risk of gallbladder cancer

Among the 20 studies on associations between gallstones and the risk of GBC, 16 studies presented associations between the presence of gallstones and the risk of GBC, as shown in Supplemental Material 1A. A total of 5 cohort studies and 11 case-control studies were included in the meta-analysis of cancer at this subsite. When we analyzed the results according to the study design, statistically significant positive associations were shown in both case-control studies (OR, 9.60; 95% CI, 4.45 to 20.70; I^2^=95.4%; p<0.001) and cohort studies (OR, 4.54; 95% CI, 2.62 to 7.87; I^2^=72.5%; p=0.006). The pooled risk estimate including case-control and cohort studies was also statistically significant (OR, 7.26; 95% CI, 4.33 to 12.18), with high heterogeneity across the studies (I^2^=93.6%; p<0.001).

Meta-analyses were stratified by diverse subgroups, as presented in Supplemenary Material 2. Regardless of the subgroups, all the results of meta-analyses were statistically significant with little differences in the magnitude of risk estimates. However, some differences in the risk estimates according to the subgroup analyses were statistically significant, as follows: geographic areas in Asia (OR, 12.72; 95% CI, 6.35 to 25.46; I^2^=86.2%; p<0.001) versus non-Asian areas (OR, 3.59; 95% CI, 2.68 to 4.81; I^2^=56.0%; p=0.026), measurement of exposure with imaging studies (OR, 15.27; 95% CI, 7.48 to 31.18; I^2^=76.9%; p=0.002) versus without imaging studies (OR, 4.67; 95% CI, 3.29 to 6.61; I^2^=76.1%; p<0.001), and adjustment for education (OR, 23.80; 95% CI, 17.00 to 33.32) versus the original summary risk estimates (OR, 7.26; 95% CI, 4.33 to 12.18; I^2^=93.6%; p<0.001).

With regard to gallstone characteristics, we found that the risk of GBC was associated with gallstone size (> 1 vs. < 1 cm: OR, 1.88; 95% CI, 1.10 to 3.22; I^2^=35.2%; p=0.201) (> 2 vs. < 2 cm: OR, 2.62; 95% CI, 0.90 to 7.60; I^2^=73.8%; p=0.022) [28,30,31,40] and gallstone number (> 1 vs. 1: OR, 2.10; 95% CI, 0.80 to 5.47; I^2^=63.8%; p=0.096) [31,40].

### Gallstones and the risk of extrahepatic bile duct cancer

A total of 17 studies presented associations between the presence of gallstones and the risk of EBDC in its broadest sense (a concept embracing EBDC, EHC, CCA, and BDC), as shown in Supplementary Material 1B. We identified 4 cohort studies and 13 casecontrol studies that presented associations between the presence of gallstones and the risk of EBDC. Among the 17 studies, 12 studies reported the risk of EBDC (or EHC), while the remaining 6 studies investigated the risk of CCA (or BDC) [23,35,37,42,44,46], with 1 study [46] describing the risk of both EBDC and CCA. The summary risk estimate for the association between gallstone presence and the risk of cancer was stronger within the studies on EBDC (or EHC) (OR, 2.87; 95% CI, 2.06 to 3.99; I^2^=95.0%; p<0.001) than the studies on CCA (or BDC) (OR, 2.12; 95% CI, 1.35 to 3.33; I^2^=92.7%; p<0.001) without statistical significance.

In the comprehensive meta-analysis of EBDC, when we analyzed the results according to the study design, a statistically significant positive association was shown in both case-control studies (OR, 3.67; 95% CI, 2.26 to 5.95; I^2^=96.0%; p<0.001) and cohort studies (OR, 2.33; 95% CI, 2.00 to 2.72; I^2^=21.4%; p=0.282). The pooled risk estimate was also statistically significant (OR, 3.17; 95% CI, 2.24 to 4.50), with high heterogeneity across the studies (I^2^=95.2%; p<0.001).

In the subgroup meta-analyses, all results showed statistical significance regardless of sex, geographic area, study period, measurement of exposure, and study quality, as presented in Supplementary Material 3. However, the differences between the magnitudes of the effect sizes did not have statistical significance in any of the stratifications.

### Gallstones and the risk of ampulla of Vater cancer

Five studies presented associations between gallstone characteristics and the risk of AOVC. Among these studies, 1 study reported the duration of gallstones [35], and all 5 studies reported the presence of gallstones [13,24,35,36,44]. Due to the limited number of eligible studies, we only conducted a meta-analysis according to the presence of gallstones, as shown in Supplementary Material 1C. The result still showed a significant association between the presence of gallstones and the risk of AOVC (OR, 3.28; 95% CI, 1.33 to 8.11; I^2^=93.3%; p<0.001). In the subgroup analyses, the magnitudes of association were significantly higher in Asian studies (OR, 7.23; 95% CI, 2.49 to 21.00; I^2^=88.0%; p=0.004) than in non-Asian studies (OR, 1.57; 95% CI, 1.28 to 1.92; I^2^=0.0%; p=0.608) and in studies that measured gallstones by an imaging modality (OR, 7.23; 95% CI, 2.49 to 21.00; I^2^=88.0%; p=0.004) than the studies that did not (OR, 1.57; 95% CI, 1.28 to 1.92; I^2^=0.0%; p=0.608) (Supplementary Material 4).

### Sensitivity analysis and publication bias

The sensitivity analyses for the relationships between the presence of gallstones and the risk of BTC are given as Supplementary Material 5. We found similar results to those of the original meta-analysis, with the same directions and magnitudes of effects (ORs ranging from 3.96 to 4.72 and each OR with a 95% CI, embodying the original OR, of 4.38) when we sequentially excluded every study one by one. The funnel plots for the association between the presence of gallstones and the risk of BTC by each subsite revealed no evidence of publication bias (Supplementary Material 6). The Egger test did not identify publication bias in the overall meta-analysis, including all the subsites of BTC (BTC: t=0.79, p=0.421; GBC: t=1.98, p=0.068; EBDC: t=0.41, p=0.688; AOVC: t=1.13, p=0.340) (Supplementary Material 6A-D).

## DISCUSSION

Our systematic review and meta-analysis provided the most comprehensive evidence to date on the associations between gallstones and the risk of BTC, including GBC, EBDC, and AOVC. This study showed that the risks of GBC, EBDC, and AOVC increased with gallstone presence, and statistically significant associations were observed both in 7 cohort studies and in 19 case-control studies. In terms of gallstone size and number, the meta-analyses revealed that only size (> 1 vs. < 1 cm) was significantly associated with the risk of GBC. Sensitivity analyses of studies restricted according to the study quality or adjustments, as well as sequentially excluding studies one by one, supported the stability of the results.

Based on the meta-analysis results for the BTC subsites, specifically GBC and AOVC, a common trend of significantly stronger summary effect sizes on the association between the presence of gallstones and the risk of cancer was present in Asian studies and studies that measured gallstones with various imaging modalities (ultrasonography, computed tomography, magnetic resonance imaging, and endoscopic retrograde cholangiopancreatography) than among their counterpart groups (Supplementary Materials 2-4). This phenomenon should be further researched to understand the reason for the difference between regions, and it underscores the importance of identifying solid evidence of gallstones in the context of a preventive approach to BTC. In addition, the summary risk estimate of GBC in accordance with gallstones’ presence, although statistically insignificant, was the strongest among the subsites of BTC (Supplementary Materials 2-4), which aligns with the settled consensus [5]. A gallbladder carrying larger gallstones or crammed with multiple gallstones is already known to increase the risk of GBC [10], and this finding was also verified through our meta-analyses (Supplementary Material 7).

The previous reports that were reviewed altogether indicated that having gallstones was associated with an increased risk of BTC [26,36,48] and each subsite of BTC: GBC [24-27,29,32,33,35-37,41,44,45,48,49], EBDC [12,24,26,32,34,36,38,39,41,43,46], and AOVC [13,24,35,36,44], although some studies reported a non-significant association [22,47]. Our study summarized the results of these studies to obtain consistent results. However, the definition of the presence of gallstones differed throughout the studies because the criteria were obscure or varied regarding the minimal required length of time between the establishment of gallstone-having status and the diagnosis of BTC. Some studies examined the presence of gallstones up to 1 year before the cancer diagnosis [44,46], while others examined the presence of gallstones up to 3 years [34,36] or more than 1 year [43] before the cancer diagnosis. Two studies [48,49] even classified a lifetime history of gallstones as the presence of gallstones. Similarly, there was a paucity of studies that reported the duration of the presence of gallstones [35], which hinders the identification of further implications on the relationships between the presence of gallstones and the carcinogenetic processes of BTC. The obscurity in definitions of the presence of gallstones and the lack of additional information on the attributes of gallstones, such as duration, may have contributed to the high heterogeneity within our meta-analyses.

With respect to the high heterogeneity of the included studies in our meta-analyses, no single factor among the study design, sex, geographic area, study period, measurement of exposure, study quality, and adjustments of confounders dramatically reduced the heterogeneity in subgroup analyses. A notable finding is that a cohort study design, male sex, and measurement of gallstones with imaging studies slightly alleviated the heterogeneity in the main analysis (Table 2). Similar trends were observed in the subsite analyses (Supplementary Mateials 2 and 3). This finding implies that the cohort studies adopting relatively objective methods for gallstone measurement reported much more precise and stable effect sizes. In the stratification by sex, wherein the degree of heterogeneity decreased in the studies that reported the outcomes of only the male group, a possible explanation may be rooted in the unique epidemiological nature of cholelithiasis and BTC, as female sex and its related attributes (sex hormones, parity, and the number of pregnancies) are well-known risk factors for both diseases [10]. Unlike male patients, female patients are impacted by additional potential confounders, which were mostly unadjusted in previous studies. Thus, determining the association between the presence of gallstones and the risk of BTC is much more complex, and the effect size of each study may tend to vary substantially.

The biological mechanisms linking gallstones to the risk of BTC are not well known. One hypothesis suggests that gallstones dropped down from the upstream biliary tract might result in chronic inflammation of the bile duct epithelium as an underlying condition for tumor development. That is, gallstones could lead to EBDC by causing inflammation of the bile duct wall [11]. In addition, approximately 35% of patients with stones develop complications such as cholecystitis or cholangitis [50], which may contribute to carcinogenesis in the gallbladder or bile ducts. Another possible hypothesis of the pathogenesis assumes that hormonal or reproductive factors might play a role in tumor development [51]. The increased exposure to endogenous estrogen and progesterone during pregnancy or exogenous estrogen seems to promote the formation of biliary stones. Under hormonal exposure, cholesterol saturation of bile mounts, leading to impaired contractility of the smooth muscles of the biliary tract [52]. Therefore, biliary stasis and gallstone formation easily occur, which might be the key steps in the process of carcinogenesis in the biliary tract [52]. Our meta-analysis results are not contradictory to either of these hypotheses.

There are several limitations to this systematic review and meta-analysis. First, we tried to capture the association between gallstones and the risk of BTC, thereby inevitably excluding some other studies [53-56] that investigated the association between gallbladder disease (or condition), not gallstones, and the risk of BTC. Second, the definition of EBDC used in our study encompassed not only EBDC and its equivalent term, EHC, but also CCA and its equivalent term, BDC. CCA (or BDC) is an overlapping term with EBDC, as approximately 90% of CCA is EBDC, but the remaining 8% to 10% comprises IBDC, which is usually not a subsite of BTC [15,57]. Third, although we extracted the risk estimates considering adjustments for potential confounders, the scope of adjusted confounders varied across the studies, which could have caused deviations in the meta-analysis results. Finally, there was significant heterogeneity across the studies, which might cast some doubts on the reliability of the summary risk estimates. This high heterogeneity may have originated from the obscurity in defining gallstones’ presence in previous studies, as most studies lacked concrete information about the duration of gallstones. This implies that the interval between the presence of gallstones and the diagnosis of BTC is inconsistent among studies, leaving the same limitations for the meta-analyses. Therefore, future research needs to implement clear criteria for gallstone presence, assuming that differences in the definition are a plausible source of heterogeneity. Another reason for this phenomenon is that our meta-analyses combined all eligible studies, which in fact, had distinct natures. In our subgroup meta-analyses, the groups that shared a common study design (cohort study), sex (male), and measurement of exposure (imaging study) showed less heterogeneity, respectively, compared to each of their counterparts. Further research with a more sophisticated approach is needed to narrow these specific groups to secure a lower level of heterogeneity when synthesizing the risk estimates on the association between gallstones and BTC.

Despite these limitations, our study has several strengths. To the best of our knowledge, this study is the first systematic review and meta-analysis of the associations between gallstone characteristics and the risk of BTC. Unlike a previous systematic review [14], we reported the characteristics of gallstones (presence, size, and number), not gallbladder disease as a whole, in association with the risk of BTC. Moreover, we conducted meta-analyses stratified by each subsite of BTC (GBC, EBDC, and AOVC) and other diverse factors, including the study design, sex, geographic area, study period, measurement of exposure, study quality, and whether analyses were adjusted for various confounders. In this study, we attempted to explore all the relevant studies and to reflect the findings and achievements hitherto established to the greatest extent possible.

## CONCLUSION

We found statistically significant associations between gallstones and an increased risk of BTC through systematic reviews and meta-analyses. We verified that the presence of gallstones is a critical risk factor for BTC as well as for GBC, EBDC, and AOVC. Our study provides a better description of the association between gallstones and the risk of BTC.

## Figures and Tables

**Table 1.** Characteristics of the studies included in the meta-analysis

Study		Country	Study period	Sample size^1^	Outcome	Gallstone characteristics	Gallstone measurement	Matching	Adjustment	NOS^2^
Cohort studies										
	Maringhini et al., 1987 [22]	USA	1950-1970	2,583 (5)	GBC	Present vs. absent	Medical records with imaging		Age, sex	7
	Bansal et al., 1996 [23]	USA	1981-1993	88,178 (104)	BDC	Present vs. absent	Medical records			5
	Chow et al., 1999 [24]	Denmark	1977-1993	17,715 (42)	GBC	Present vs. absent	Medical records		Age, sex	8
				(12)	EBDC	Present vs. absent				
				(8)	AOVC	Present vs. absent				
	Zou et al., 2000 [25]	China	1986-1998	105,019 (3,922)	GBC	Present vs. absent	Medical records			6
	Ishiguro et al., 2008 [26]	Japan	1990-1994	101,868 (235)	BTC	Present vs. absent	Self-reported		Age, sex, area, history of gallstone disease, BMI, DM, alcohol, smoking	5
				(93)	GBC	Present vs. absent				
				(142)	EBDC	Present vs. absent				
	Nordenstedt et al., 2012 [12]	Sweden	1965-2008	192,960 (169)	EBDC	Present vs. absent	Medical records		Age, sex, calendar year	8
	Lai et al., 2013 [27]	Taiwan	1996-2008	DM	GBC	Present vs. absent	Medical records		Age, sex, hypertension, hyperlipidemia, obesity	7
				214,179 (206)						
				Non-DM						
				206,860 (147)						
Case-control studies										
	Diehl, 1983 [28]	USA	1976-1980	45/66	GBC	Size (≥1 vs. <1)	Medical records	Age, sex, hospital, screening year		7
						Size (≥2 vs. <2)				
	Lowenfels et al., 1985 [29]	USA	Black 1965-1978/	74/2,013	GBC	Present vs. absent	Medical records		Age	5
			White 1977-1983/							
			Indian 1963-1983	57/386	GBC	Present vs. absent				
	Lowenfels et al., 1989 [30]	USA	1979-1985	15/398	GBC	Size (≥1 vs. <1)	Medical records		Age, race	7
						Size (≥2 vs. <2)				
	Moerman et al., 1993 [31]	Netherlands	1966-1969	43/98	GBC	Size (≥1 vs. <1)	Medical records with imaging	Age, sex, hospital, date of admission		7
				41/96	GBC	Size (≥2 vs. <2)				
						n (>1 vs 1)				
	Khan et al., 1999 [32]	USA	1980-1994	38/138	GBC	Present vs. absent	Medical records		Age, sex, ethnicity, socioeconomic status, smoking	6
				31/138	EBDC	Present vs. absent				
	Okamoto et al., 1999 [33]	Japan	1986-1993	19/194,478	GBC	Present vs. absent	Medical records with imaging			6
	Welzel et al., 2007 [34]	USA	1993-1999	549/102,782	EHC	Present vs. absent^3^	Medical records		Age, sex, race, geographic region, state buy-in status	6
	Hsing et al., 2007 [35]	China	1997-2001	368/902	GBC	Present vs. absent	Medical records with imaging	Age	Age, sex, education	8
						Duration (≥10 vs. <10)				
				191/959	BDC	Present vs. absent				
						Duration (≥10 vs. <10)				
				68/959	AOVC	Present vs. absent				
						Duration (≥10 vs. <10)				
	Ahrens et al., 2007 [36]	Europe	1995-1997	153/1,421	BTC	Present vs. absent^3^	Self-reported	Age, sex, region	Age, country, next-of-kin status	7
				(45)	GBC	Present vs. absent^3^				
				(52)	EBDC	Present vs. absent^3^				
				(47)	AOVC	Present vs. absent^3^				
	Grainge et al., 2009 [37]	UK	1987-2002	372/5,760	CCA	Present vs. absent	Medical records	Sex, age, GP practice	Alcohol, smoking, BMI	5
				184/5,760	GBC	Present vs. absent				
	Tao et al., 2010 [38]	China	1998-2008	129/380	EHC	Present vs. absent	Medical records with imaging	Age, sex	Age, sex, DM, history of cholecystectomy	8
	Cai et al., 2011 [39]	China	2000-2005	313/608	EHC	Present vs. absent	No records	Age, sex	Age, sex	7
	Alvi et al., 2011 [40]	Pakistan	1988-2007	60/120	GBC	Size (≥1 vs. <1)	Medical records with imaging		Age, parity, BMI, stone characteristics	5
						n (>1 vs. 1)				
	Wu et al., 2012 [41]	China	1998-2010	93/809	GBC	Present vs. absent	Medical records with imaging	Age, sex	Age, sex, HBV, DM, TC, HDL-C	9
				86/835	EBDC	Present vs. absent				
	Onal et al., 2012 [42]	Turkey	2006-2010	99/48	CCA	Present vs. absent	Self-reported	Age, sex	Age, sex, HBV, alcohol, smoking	6
	Chang et al., 2013 [43]	Taiwan	2004-2008	2,179/8,716	EHC	Present vs. absent^4^	Medical records with imaging	Age, sex, date of diagnosis	Cholangitis	5
	Nogueira et al., 2014 [44]	USA	1992-2005	3,681/100,000	CCA	Present vs. absent^5^	Medical records	Age, sex, calendar year	Age, sex, calendar year	6
				(3,664)	GBC	Present vs. absent^5^				
				(1,646)	AOVC	Present vs. absent^5^				
	He et al., 2014 [13]	China	2006-2010	210/620	AOVC	Present vs. absent	Medical records with imaging	Age, sex		8
	Cha, 2015 [45]	Korea	2008-2013	78/78	GBC	Present vs. absent	Medical records with imaging	Age, sex	Hypertension, DM, VOD, alcohol, smoking, BMI, PLG	8
	Lee et al., 2015 [46]	Korea	2007-2013	276/552	CCA	Present vs. absent^5^	Medical records	Age, sex, date of diagnosis	Alcohol, DM, HBV, LFI	8
				193/386	EBDC	Present vs. absent^5^				
	Lee et al., 2015 [47]	Korea	2007-2013	81/162	EHC	Present vs. absent	Medical records	Age, sex, date of diagnosis	DM, smoking	8
	Rosato et al., 2016 [48]	Italy	Study 1: 1983-1992	159/795	BTC	Present vs. absent^6^	Self-reported	Age, sex, study, center	Year of interview, education, BMI, alcohol, smoking	8
			Study 2: 1994-2009		GBC	Present vs. absent^6^				
	Tamrakar et al., 2016 [49]	Nepal	2012-2013	100/100	GBC	Present vs. absent^6^	Self-reported	Age, sex, marital status	Education, hospital, smoking, fruit consumption, residence	6

NOS, Newcastle-Ottawa scale; AOVC, ampulla of Vater cancer; BDC, bile duct cancer; BMI, body mass index; BTC, biliary tract cancer; CCA, cholangiocarcinoma; DM, diabetes mellitus; EBDC, extrahepatic bile duct cancer; EHC, extrahepatic cholangiocarcinoma; GBC, gallbladder cancer; GP, general physician; HBV, hepatitis B virus; TC, total cholesterol; HDL-C, high density lipoprotein cholesterol; LFI, liver fluke infestation; PLG, polypoid lesion of gallbladder; VOD, vascular occlusive disease.

1 Number of participants (cases) for cohort studies and cases/controls (cases of subsites) for case-control studies.

2 NOS for assessing the quality of non-randomized studies in a meta-analysis.

3 Gallstone presence of up to 3 years before the diagnosis of cancer.

4 Gallstone presence more than 1 year before the diagnosis of cancer.

5 Gallstone presence of up to 1 year before the diagnosis of cancer.

6 History of gallstones (ever vs. never).

**Table 2.** Meta-analysis results for the association between the presence of gallstones and the risk of BTC by subgroups

Subgroup	No. of studies	OR (95% CI)^1^	I^2^ value (%)	p for heterogeneity
All studies	26	4.38 (3.23, 5.93)	91.2	<0.001
Study design				
Cohort study	7	3.17 (2.28, 4.39)	79.0	<0.001
Case-control study	19	5.04 (3.36, 7.56)	90.5	<0.001
Sex				
Male	9	3.40 (2.70, 4.28)	35.8	0.132
Female	9	4.26 (2.75, 6.59)	84.5	<0.001
Geographic area				
Asia	15	5.25 (3.50, 7.86)	82.4	<0.001
Non-Asia^2^	11	3.58 (2.17, 5.91)	95.1	<0.001
Study period^3^				
Before 2000	8	5.39 (2.57, 11.34)	95.5	<0.001
Around 2000	7	2.67 (2.10, 3.39)	38.6	0.135
After 2000	7	5.21 (2.13, 12.74)	85.7	<0.001
No records	4	5.73 (2.61, 12.61)	87.3	<0.001
Measurement of gallstones				
Medical records with imaging studies	8	7.09 (3.87, 12.98)	64.5	0.004
Medical records without imaging studies	16	3.81 (2.48, 5.85)	93.9	<0.001
No records	2	3.47 (2.88, 4.18)	17.1	0.272
Study quality^4^				
High NOS	14	3.99 (2.85, 5.59)	75.6	<0.001
Low NOS	12	4.81 (2.87, 8.05)	94.9	<0.001
Adjustment for age, yes	21	3.71 (2.66, 5.16)	90.6	<0.001
Adjustment for sex, yes	20	3.92 (2.77, 5.55)	91.2	<0.001
Adjustment for comorbidities, yes	7	3.05 (1.84, 5.05)	75.7	<0.001
Adjustment for lifestyle factors, yes^5^	7	4.84 (1.95, 11.98)	85.5	<0.001
Adjustment for education, yes	1	9.42 (3.56, 24.91)	-	-
Adjustment for geographic areas, yes	4	7.34 (2.28, 23.62)	87.6	0.000

BTC, biliary tract cancer; OR, odds ratio; CI, confidence interval; NOS, Newcastle-Ottawa scale.

1 OR refers to a summary estimate of effects based on a random-effects model.

2 Non-Asia including USA and European areas.

3 Study period was defined by the study’s starting point (a) and ending point (b). Before 2000, (a) and (b) are both before 2000; around 2000, (a) is before 2000 but (b) is after 2000; after 2000, (a) and (b) are both after 2000.

4 Quality scores greater than or equal to the median value were judged as a high NOS (≥7).

5 Adjustment for lifestyle factors such as alcohol, smoking, body mass index, etc.
