# Supplementary material for: Association between gallstones and the risk of biliary tract cancer: a systematic review and meta-analysis
Source: Epidemiol Health. 2021 Feb 3;43:e2021011. doi: 10.4178/epih.e2021011 (PMC8060519; doi:10.4178/epih.e2021011)
Supplement: Supplementary Material 1. [file epih-43-e2021011-suppl1.pdf]

Volume: 43, Article ID: e2021011

https://doi.org/10.4178/epih.e2021011

SUPPLEMENTARY MATERIALS

(A)

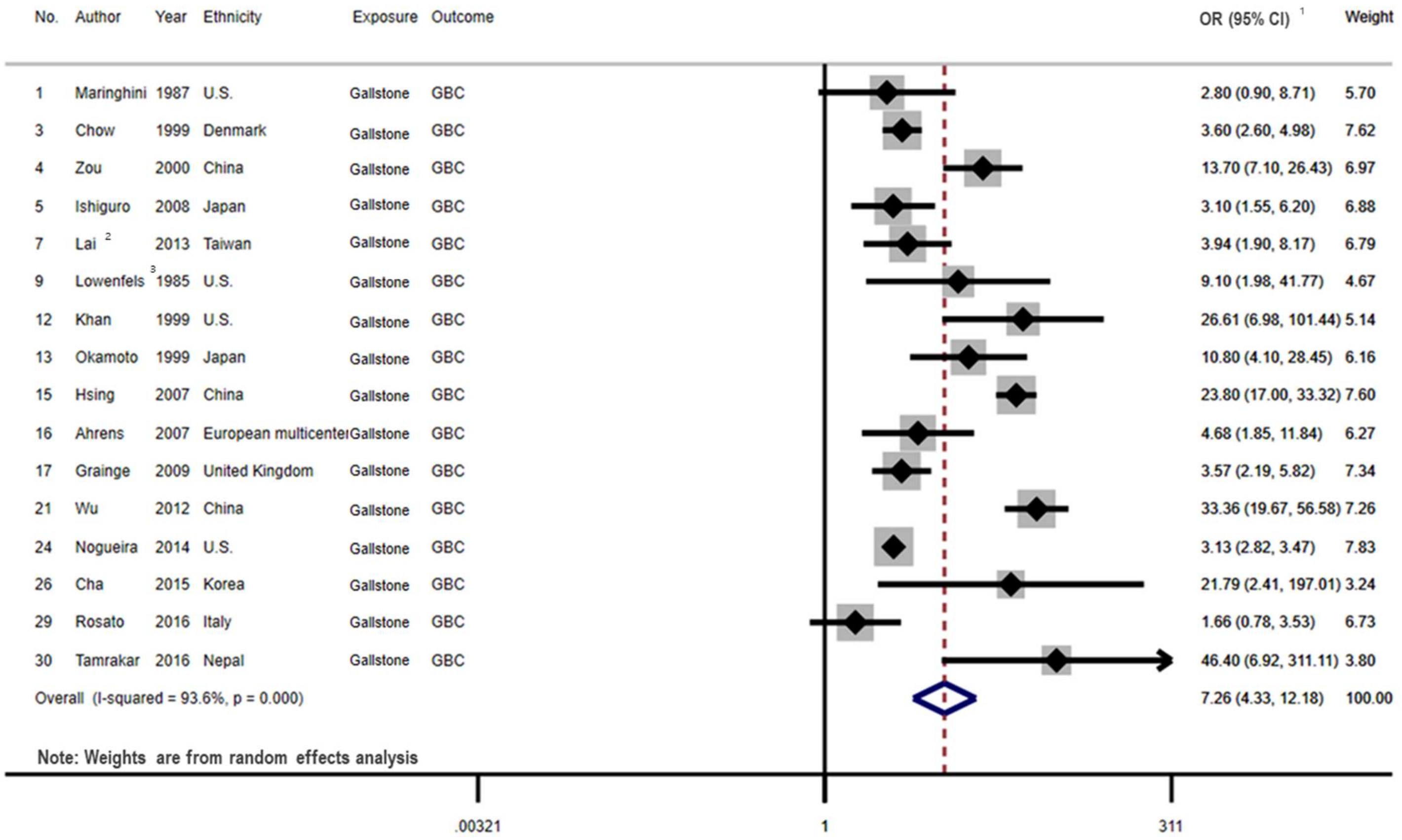

Volume: 43, Article ID: e2021011

https://doi.org/10.4178/epih.e2021011

(B)

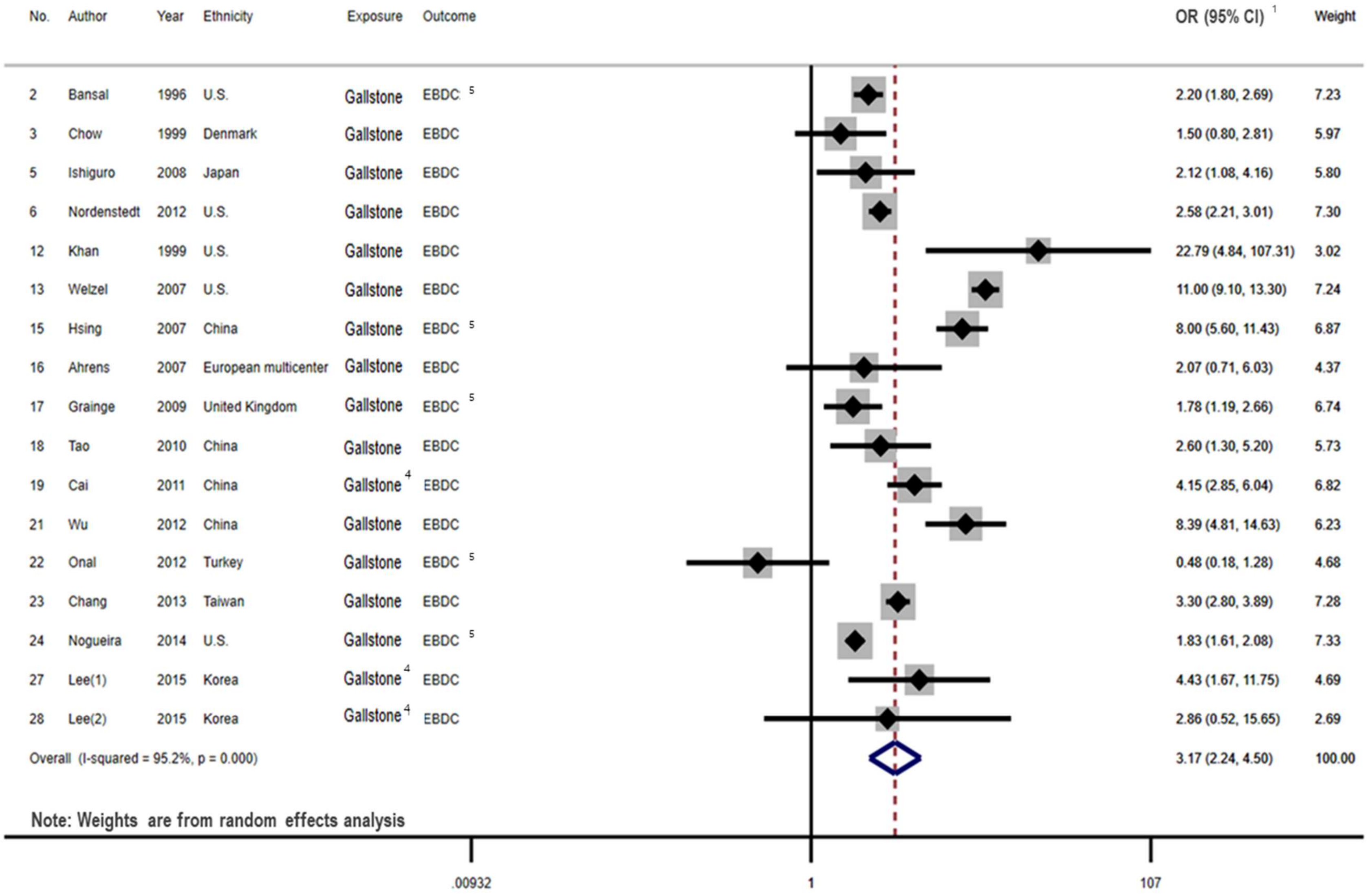

Volume: 43, Article ID: e2021011

https://doi.org/10.4178/epih.e2021011

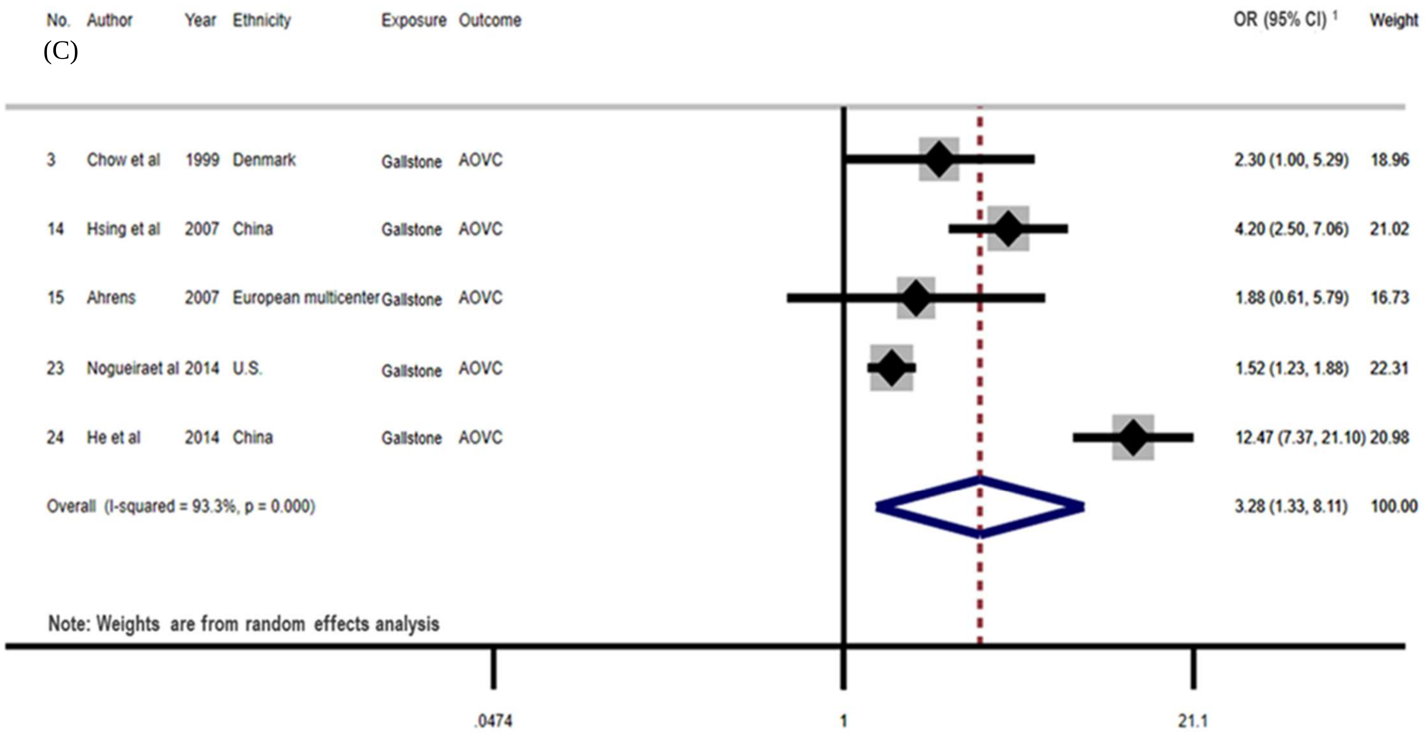

Supplementary Material 1. Forest plot showing the relationship between presence of gallstone and the risk of (A) GBC, (B) EBDC, and (C) AVOC. [1]OR (95% CI) refers to the estimate of effects included in a random effects model. [2]Effect size of Lai et al. [27] was calculated by pooling the results of DM group and non-DM group. [3] Effect size of Lowenfels et al. [29] was calculated by pooling the results of indian and non-indian. [4]Pooled the risk estimates of cholecystolithiasis and choledocholithiasis group. [5]Pooled the risk estimates of CCA and EBDC group. The numbers are arranged in the order of Table1. OR, odds ratio; CI, confidence interval; GBC, gallbladder cancer; BTC, biliary tract cancer; EBDC, extrahepatic bile duct cancer; AOVC, ampulla of Vater cancer.
