# Supplementary material for: Association between gallstones and the risk of biliary tract cancer: a systematic review and meta-analysis
Source: Epidemiol Health. 2021 Feb 3;43:e2021011. doi: 10.4178/epih.e2021011 (PMC8060519; doi:10.4178/epih.e2021011)
Supplement: Supplementary Material 2. [file epih-43-e2021011-suppl2.pdf]

Volume: 43, Article ID: e2021011

https://doi.org/10.4178/epih.e2021011

Supplementary Material 2. Meta-analysis results for association between the presence of gallstone and the risk of GBC by the subgroups

Subgroup	No. of study	OR (95%CI) [1]	I^2 value (%)	P for heterogeneity
All studies	16	7.26 (4.33-12.18)	93.6	<0.001
Study design				
Cohort study	5	4.54 (2.62-7.87)	72.5	0.006
Case-control study	11	9.60 (4.45-20.70)	95.4	<0.001
Sex				
Male	6	6.69 (3.13-14.31)	74.8	0.001
Female	6	6.69 (2.46-18.15)	87.4	<0.001
Geographic area				
Asia	8	12.72 (6.35-25.46)	86.2	<0.001
Non-Asia [2]	8	3.59 (2.68-4.81)	56.0	0.026
Study period [3]				
Before 2000	6	6.22 (3.24-11.95)	57.3	0.039
Around 2000	5	4.54 (1.75-11.81)	97.0	<0.001
After 2000	2	33.59 (7.96-141.72)	0.0	0.611
No record	3	11.66 (2.70-50.45)	96.3	0.000
Measure of gallstone				
Medical record with imaging study	5	15.27 (7.48-31.18)	76.9	0.002
Medical record without imaging study	11	4.67 (3.29-6.61)	76.1	<0.001
Study quality [4]				
High NOS	8	6.72 (2.80-16.10)	94..0	<0.001
Low NOS	8	7.25 (4.08-12.88)	83.8	<0.001
Adjustment for age, yes	12	6.90 (3.80-12.51)	94.6	<0.001

Volume: 43, Article ID: e2021011

https://doi.org/10.4178/epih.e2021011

Adjustment for sex, yes	11	7.83 (4.11-14.91)	95.2	<0.001
Adjustment for comorbidities, yes	3	3.99 (2.14-7.45)	27.5	0.252
Adjustment for lifestyle factors, yes [5]	6	6.87 (3.36-14.04)	69.9	0.005
Adjustment for education, yes	1	23.80 (17.00-33.32)	-	-
Adjustment for geographic areas, yes	4	9.32 (2.95-29.37)	76.7	0.005

GBC, gallbladder cancer; OR, odds ratio; NOS, Newcastle-Ottawa scale.

[1] OR (Odds ratio) refers to summary estimate of effects based on random effects model. [2] Non-Asia including U.S. and European areas. [3] Study period is defined by the study's starting point (a) and ending point (b). Before 2000, (a) and (b) are both before 2000; Around 2000, (a) is before 2000 but (b) is after 2000; After 2000, (a) and (b) are both after 2000. [4] The quality scores equal or more than the median value was judged as high NOS (≥7). [5] Adjustment for lifestyle factors such as alcohol, smoking, BMI, or etc..
