# Supplementary material for: Association between gallstones and the risk of biliary tract cancer: a systematic review and meta-analysis
Source: Epidemiol Health. 2021 Feb 3;43:e2021011. doi: 10.4178/epih.e2021011 (PMC8060519; doi:10.4178/epih.e2021011)
Supplement: Supplementary Material 3. [file epih-43-e2021011-suppl3.pdf]

Volume: 43, Article ID: e2021011

https://doi.org/10.4178/epih.e2021011

Supplementary Material 3. Meta-analysis results for association between presence of gallstone and the risk of EBDC by subgroups

Subgroup	No. of study	OR (95%CI) [1]	I^2 value (%)	P for heterogeneity
All studies	17	3.17 (2.24-4.50)	95.2	<0.001
Subsite				
EBDC (or EHC)	12 [2]	2.87 (2.06-3.99)	95.0	<0.001
CCA (or BDC)	6 [2]	2.12 (1.35-3.33)	92.7	<0.001
Study design				
Cohort study	4	2.33 (2.00-2.72)	21.4	0.282
Case-control study	13	3.67 (2.26-5.95)	96.0	<0.001
Sex				
Male	5	3.46 (2.29-5.22)	78.6	0.001
Female	5	5.13 (2.73-9.66)	91.2	<0.001
Geographic area				
Asia	9	3.48 (2.30-5.28)	83.6	<0.001
Non-Asia 3	8	2.99 (1.72-5.19)	97.4	<0.001
Study period [4]				
Before 2000	5	4.43 (1.61-12.20)	97.2	<0.001
Around 2000	5	2.79 (1.80-4.32)	93.6	<0.001
After 2000	4	2.28 (0.79-6.55)	82.0	0.001
No record	3	3.50 (1.66-7.35)	88.1	<0.001
Measure of gallstone				
Medical record with imaging study	4	5.52 (3.07-9.93)	69.1	0.021
Medical record without imaging study	11	2.60 (1.61-4.20)	96.4	<0.001
No record	2	3.47 (2.88-4.18)	17.1	0.272
Study quality [5]				

Volume: 43, Article ID: e2021011

https://doi.org/10.4178/epih.e2021011

High NOS	9	3.64 (2.37-5.59)	84.9	<0.001
Low NOS	8	2.79 (1.58-4.93)	97.5	<0.001
Adjustment for age, yes	16	3.26 (2.23-4.78)	95.3	<0.001
Adjustment for sex, yes	15	3.34 (2.26-4.94)	95.6	<0.001
Adjustment for comorbidities, yes	5	2.60 (1.39-4.89)	85.6	<0.001
Adjustment for lifestyle factors, yes [6]	4	2.12 (0.83-5.44)	82.9	0.001
Adjustment for education, yes	1	8.00 (5.60-11.43)	-	-
Adjustment for geographic areas, yes	3	3.92 (1.16-13.29)	75.0	0.018

BDC, bile duct cancer; CCA, cholangiocarcinoma; EBDC, extrahepatic bile duct cancer; EHC, extrahepatic cholangiocarcinoma; OR, odds ratio; NOS, Newcastle-Ottawa Scale.

[1] OR (Odds ratio) refers to summary estimate of effects based on random effects model. [2] one study [30] suggested the risk estimates of both EBDC and CCA. [3] Non-Asia including U.S. and European areas. [4] Study period is defined by the study's starting point (a) and ending point (b). Before 2000, (a) and (b) are both before 2000; Around 2000, (a) is before 2000 but (b) is after 2000; After 2000, (a) and (b) are both after 2000. [5] The quality score equal or more than median value was judged as high NOS (≥7). [6] Adjustment for lifestyle factors such as alcohol, smoking, BMI, or etc.
