# Supplementary material for: Association between gallstones and the risk of biliary tract cancer: a systematic review and meta-analysis
Source: Epidemiol Health. 2021 Feb 3;43:e2021011. doi: 10.4178/epih.e2021011 (PMC8060519; doi:10.4178/epih.e2021011)
Supplement: Supplementary Material 4. [file epih-43-e2021011-suppl4.pdf]

Volume: 43, Article ID: e2021011

https://doi.org/10.4178/epih.e2021011

Supplementary Material 4. Meta-analysis results for association between presence of gallstone and the risk of AOVC by subgroups

Subgroup	No. of study	OR (95%CI)[1 *1]	I^2 value (%)	*P* for heterogeneity
All studies	5	3.28 (1.33-8.11)	93.3	<0.001
Study design				
Cohort study	1	2.30 (1.00-5.29)	-	-
Case-control study	4	3.56 (1.20-10.54)	95.0	<0.001
Sex				
Male	1	3.60 (1.70-7.62)	-	-
Female	2	3.30 (1.41-7.70)	43.2	0.185
Ethnicity				
Asia	2	7.23 (2.49-21.00)	88.0	0.004
Non-Asia [2]	3	1.57 (1.28-1.92)	0.0	0.608
Study period [3]				
Before 2000	1	1.88 (0.61-5.79))	-	-
Around 2000	2	2.46 (0.91-6.61)	92.1	<0.001
After 2000	1	12.47 (7.37-21.10)	-	-
No record	1	2.30 (1.00-5.29)	-	-
Measure of gallstone				
Medical record with imaging	2	7.23 (2.49-21.00)	88.0	0.004
Medical record without imaging	3	1.57 (1.28-1.92)	0.0	0.608
Study quality [4]				

Volume: 43, Article ID: e2021011

https://doi.org/10.4178/epih.e2021011

High NOS	4	4.18 (1.79-9.80)	83.4	<0.001
Low NOS	1	1.52 (1.23-1.88)	-	-
Adjustment for age, yes	4	2.29 (1.26-4.17)	77.0	0.005
Adjustment for sex, yes	3	2.39 (1.17-4.91)	84.6	0.001
Adjustment for education, yes	1	4.20 (2.50-7.06)	-	-

AOVC, ampulla of Vater cancer; OR, odds ratio; NOS, Newcastle-Ottawa Scale.

[1] OR (Odds ratio) refers to summary estimate of effects based on random effects model. [2] Non-Asia including U.S. and European areas. [3] Study period is defined by the study's starting point (a) and ending point (b). Before 2000, (a) and (b) are both before 2000; Around 2000, (a) is before 2000 but (b) is after 2000; After 2000, (a) and (b) are both after 2000. [4] The quality score equal or more than median value was judged as high NOS (≥7).
