# Supplementary material for: Association between gallstones and the risk of biliary tract cancer: a systematic review and meta-analysis
Source: Epidemiol Health. 2021 Feb 3;43:e2021011. doi: 10.4178/epih.e2021011 (PMC8060519; doi:10.4178/epih.e2021011)
Supplement: Supplementary Material 5. [file epih-43-e2021011-suppl5.pdf]

Volume: 43, Article ID: e2021011

https://doi.org/10.4178/epih.e2021011

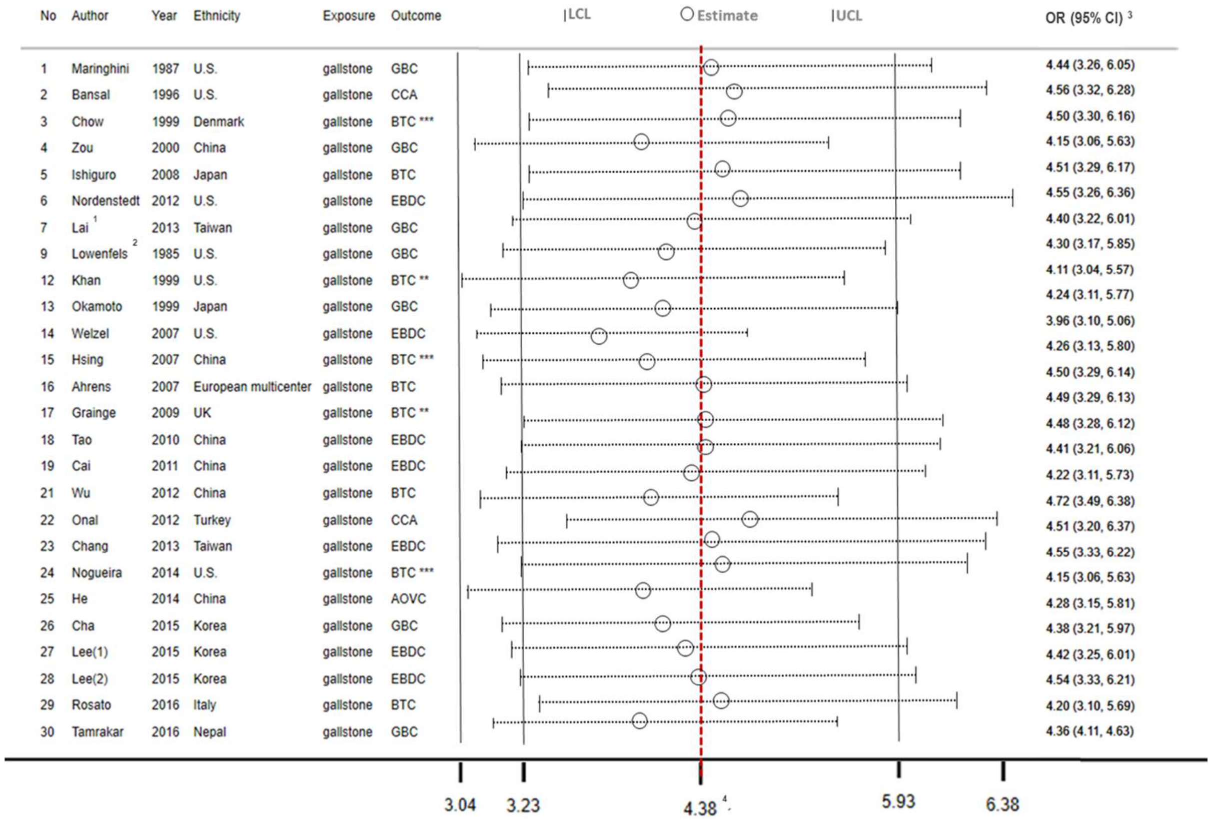

Supplementary Material 5. Sensitivity analysis showing the relationship between gallstones and the BTC risk

[1] Effect size of Lai et al. was calculated by pooling the results of DM group and non-DM group.

[2] Effect size of Lowenfels et al. was calculated by pooling the results of indian and non-indian group.

[3] OR (Odds ratio) and 95% CI (Confidence interval) refers to the summary estimate of effects in a random effects model if each one of the study is excluded.

[4] Summary risk estimate of effects in a random effects model where all the study is included

** Pooled the results of two types BTC subsites.

*** Pooled the results of three types BTC subsites.
