# Supplementary material for: Association between gallstones and the risk of biliary tract cancer: a systematic review and meta-analysis
Source: Epidemiol Health. 2021 Feb 3;43:e2021011. doi: 10.4178/epih.e2021011 (PMC8060519; doi:10.4178/epih.e2021011)
Supplement: Supplementary Material 6. [file epih-43-e2021011-suppl6.pdf]

Volume: 43, Article ID: e2021011

https://doi.org/10.4178/epih.e2021011

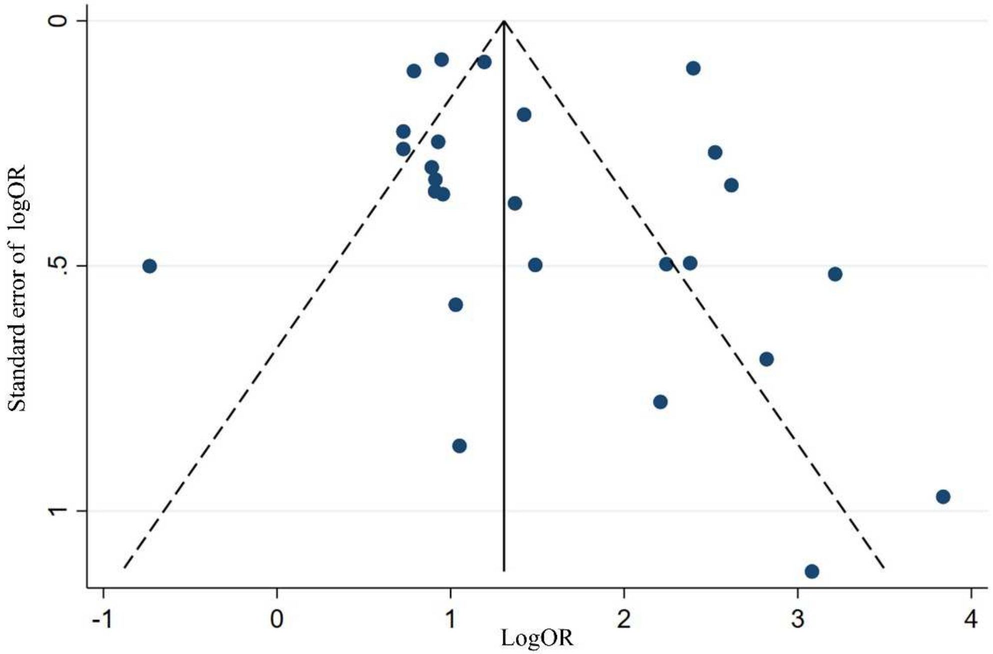

Supplementary Material 6A. Funnel plot showing the relationship between gallstone and the BTC risk.
Pseudo 95% confidence limits indicated as dotted line. Egger's regression asymmetry test (BTC, t=0.79, p=0.421).

Volume: 43, Article ID: e2021011

https://doi.org/10.4178/epih.e2021011

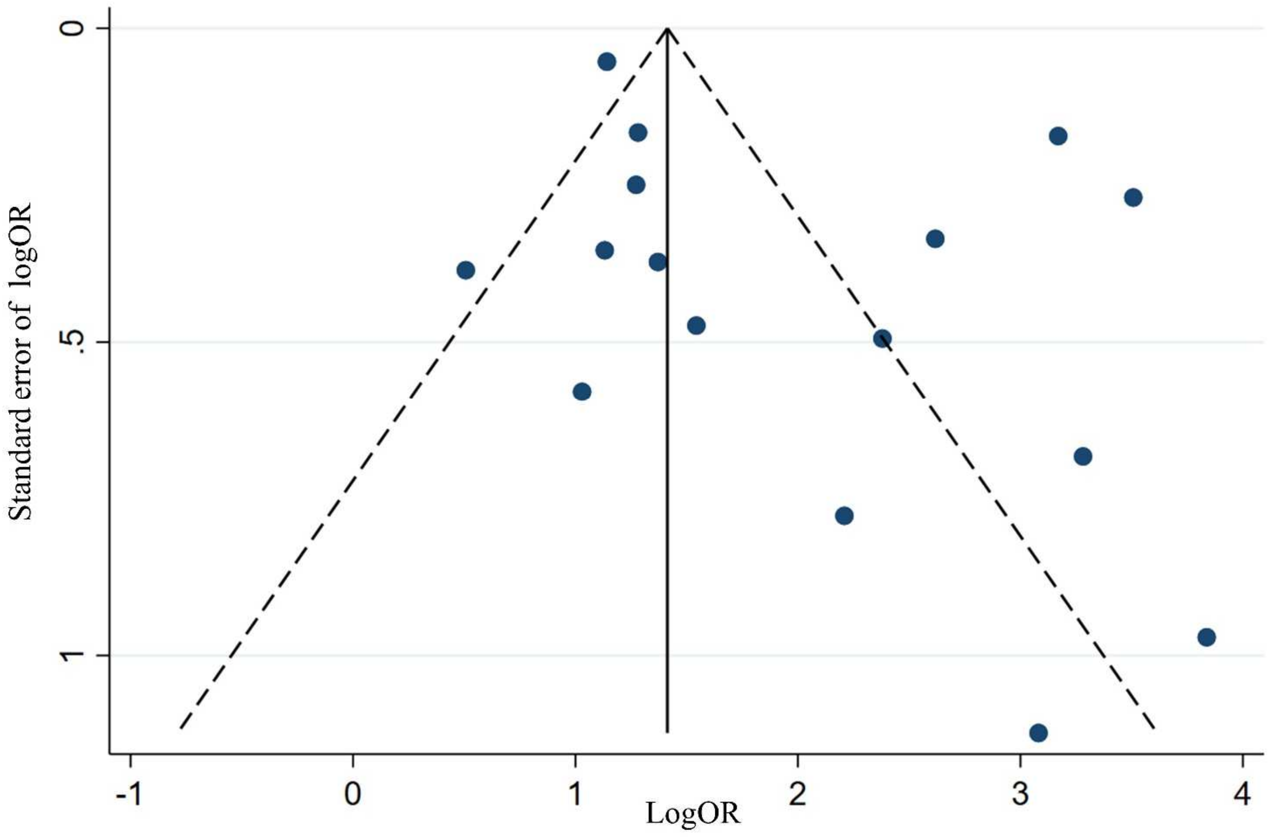

Supplementary Material 6B. Funnel plot showing the relationship between gallstone and the GBC risk.
Pseudo 95% confidence limits indicated as dotted line. Egger's regression asymmetry test (GBC, t=1.98, p=0.068).

Volume: 43, Article ID: e2021011

https://doi.org/10.4178/epih.e2021011

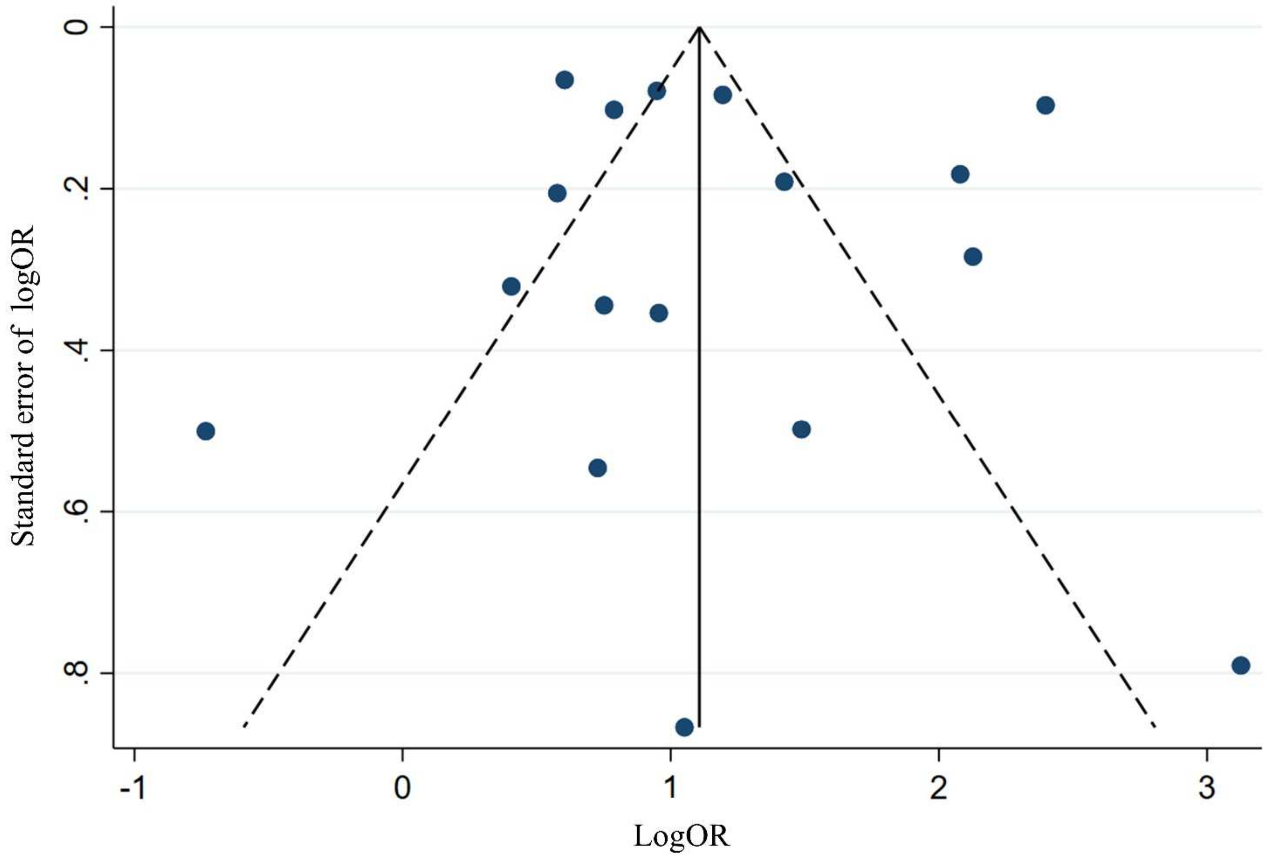

Supplementary Material 6C. Funnel plot showing the relationship between gallstone and the EBDC risk.
Pseudo 95% confidence limits indicated as dotted line. Egger's regression asymmetry test (EBDC, t=0.41, p=0.688).

Volume: 43, Article ID: e2021011

https://doi.org/10.4178/epih.e2021011

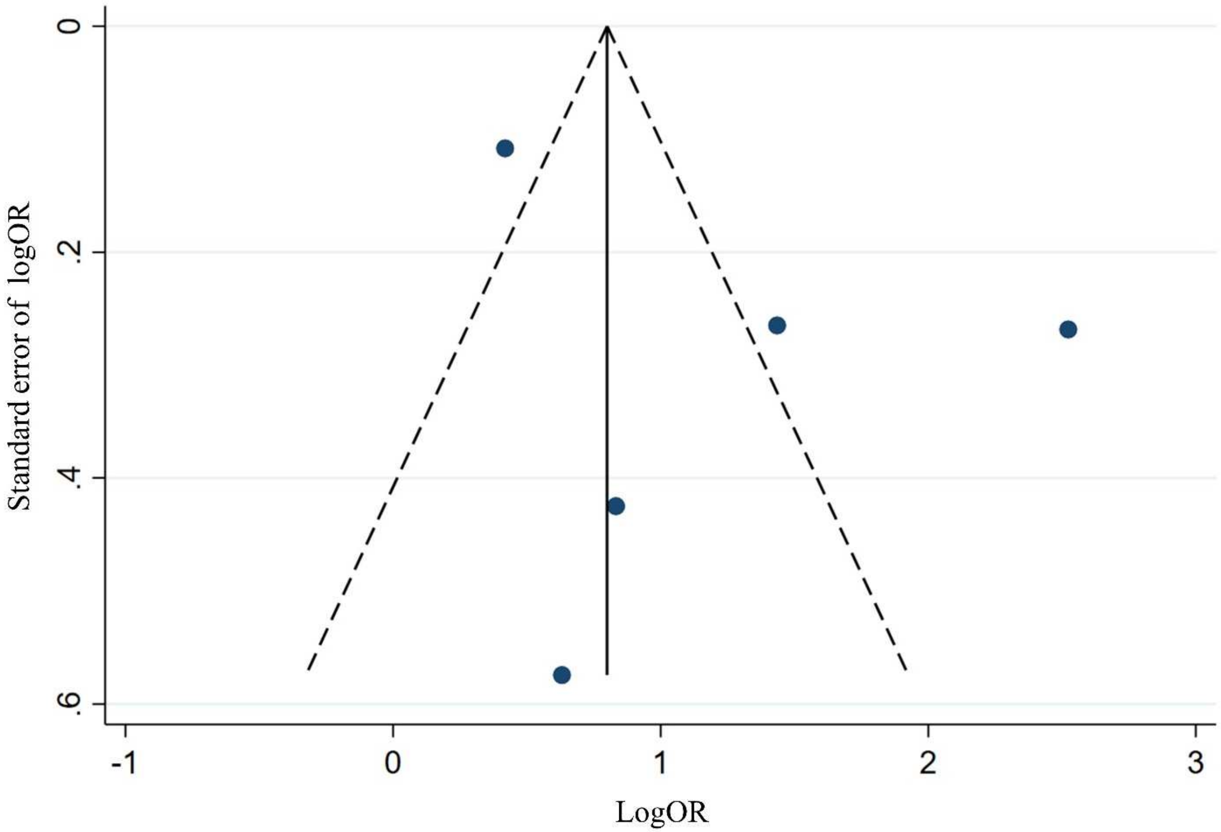

Supplementary Material 6D. Funnel plot showing the relationship between gallstone and the AOVC risk.
Pseudo 95% confidence limits indicated as dotted line. Egger's regression asymmetry test (AOVC, t=1.13, p=0.340).
