# Supplementary material for: Association between gallstones and the risk of biliary tract cancer: a systematic review and meta-analysis
Source: Epidemiol Health. 2021 Feb 3;43:e2021011. doi: 10.4178/epih.e2021011 (PMC8060519; doi:10.4178/epih.e2021011)
Supplement: Supplementary Material 7. [file epih-43-e2021011-suppl7.pdf]

Volume: 43, Article ID: e2021011

https://doi.org/10.4178/epih.e2021011

Supplementary Material 7. Systematic review and meta-analysis results for association between gallstone characteristics and the risk of BTC

Characteristics	No. of study	OR (95%CI) [1]	I^2 value (%)	*P* for heterogeneity
Size (≥1cm vs. <1cm)	4	1.88 (1.10-3.22)	35.2	0.201
Size (≥2cm vs. <2cm)	3	2.62 (0.90-7.60)	73.8	0.022
Number (>1 vs. 1)	2	2.10 (0.80-5.47)	63.8	0.096

Abbreviations: BTC (biliary tract cancer), OR (odds ratio).

[1] OR refers to summary estimate of effects based on random effects model.
